# Geographic disparities and temporal changes of COVID-19 incidence risks in North Dakota, United States

**DOI:** 10.1186/s12889-023-15571-5

**Published:** 2023-04-20

**Authors:** Nirmalendu Deb Nath, Md Marufuzzaman Khan, Matthew Schmidt, Grace Njau, Agricola Odoi

**Affiliations:** 1 Department of Biomedical and Diagnostic Sciences, College of Veterinary Medicine, University of Tennessee, Knoxville, TN USA; 2 Department of Public Health, College of Education, Health, and Human Sciences, University of Tennessee, Knoxville, TN USA; 3 North Dakota Department of Health and Human Services, Special Projects and Health Analytics, Bismarck, ND USA

**Keywords:** COVID-19, Spatial epidemiology, Geographic disparities, Geographic information system, FlexScan, North Dakota

## Abstract

**Background:**

COVID-19 is an important public health concern due to its high morbidity, mortality and socioeconomic impact. Its burden varies by geographic location affecting some communities more than others. Identifying these disparities is important for guiding health planning and service provision. Therefore, this study investigated geographical disparities and temporal changes of the percentage of positive COVID-19 tests and COVID-19 incidence risk in North Dakota.

**Methods:**

COVID-19 retrospective data on total number of tests and confirmed cases reported in North Dakota from March 2020 to September 2021 were obtained from the North Dakota COVID-19 Dashboard and Department of Health, respectively. Monthly incidence risks of the disease were calculated and reported as number of cases per 100,000 persons. To adjust for geographic autocorrelation and the small number problem, Spatial Empirical Bayesian (SEB) smoothing was performed using queen spatial weights. Identification of high-risk geographic clusters of percentages of positive tests and COVID-19 incidence risks were accomplished using Tango’s flexible spatial scan statistic. ArcGIS was used to display and visiualize the geographic distribution of percentages of positive tests, COVID-19 incidence risks, and high-risk clusters.

**Results:**

County-level percentages of positive tests and SEB incidence risks varied by geographic location ranging from 0.11% to 13.67% and 122 to 16,443 cases per 100,000 persons, respectively. Clusters of high percentages of positive tests were consistently detected in the western part of the state. High incidence risks were identified in the central and south-western parts of the state, where significant high-risk spatial clusters were reported. Additionally, two peaks (August 2020-December 2020 and August 2021-September 2021) and two non-peak periods of COVID-19 incidence risk (March 2020-July 2020 and January 2021-July 2021) were observed.

**Conclusion:**

Geographic disparities in COVID incidence risks exist in North Dakota with high-risk clusters being identified in the rural central and southwest parts of the state. These findings are useful for guiding intervention strategies by identifying high risk communities so that resources for disease control can be better allocated to communities in need based on empirical evidence. Future studies will investigate predictors of the identified disparities so as to guide planning, disease control and health policy.

**Supplementary Information:**

The online version contains supplementary material available at 10.1186/s12889-023-15571-5.

## Introduction

Coronavirus disease 2019 (COVID-19) was declared a pandemic on 11 March 2020 by World Health Organization [1], and since then United States (US) has reported the highest number of confirmed cases and deaths as of 22 March 2022 [2]. The state of North Dakota (ND) identified the first COVID-19 case on 11 March 2020, which was followed by an upsurge in the number of cases in all counties in the state resulting in significant impact to both the health and economic well being of the state [3]. Therefore, the governor of ND declared COVID-19 pandemic a major disaster on 29 March same year [4] with the state reporting a total of 239,672 positive cases with 2,242 deaths by 22 March 2022 [2].

There is evidence that the incidence and severity of COVID-19 vary by geographical region due, at least in part, to differences in population characteristics such as socio-economic, demographic, and chronic health conditions [5–8]. For example, previous studies have reported higher COVID-19 incidence risks in geographic regions with a high proportion of Black/or Hispanic individuals [9–11]. The American Public Media Research Lab reported that the COVID-19 mortality rate was 2.3 times higher in Black people as compared with White Americans [12]. Other social determinants, including access to healthcare, income inequality, high population density, and cultural beliefs may influence disease incidence and burden. In addition, certain occupations (e.g. doctors, nurses, laboratory professionals, road workers) may also be at higher risks of the disease [13–15].

Population differences in levels of mobility is another factor that might contribute to geographic differences in COVID-19 risks. Previous studies found high correlations between mobility and the COVID-19 burden in counties of China and the US [16, 17]. Chang and his co-workers reported that the COVID-19 infection rates were higher among low socio-economic groups [18] than higher income groups. This is attributed to the fact that individuals in low-income brackets tend to work in more crowded environments and are more likely to use public transport system. Therefore, they are more likely to be exposed to COVID-19 resulting in higher disease incidence in these populations [18]. Moreover, low-income populations tend to experience household overcrowding which also increases risk of exposure to COVID-19 [10, 19, 20].

Identifying geographical disparities of COVID-19 risk is important for guiding health planning and policies for disease control and prevention. Unfortunately, very little is known about geographic disparities of COVID-19 risk in ND. Therefore, the objective of this study was to identify geographic disparities and temporal changes of percentage of positive COVID-19 tests and COVID-19 incidence risk in ND to guide control efforts.

## Methods

### Ethics approval

This study was approved by the University of Tennessee Institutional Review Board (IRB number: UTK IRB-22–07032-XM) and all study methods were carried out in accordance with relevant guidelines and regulations. The investigators did not contact the subjects.

### Study area

**Fig. 1:**
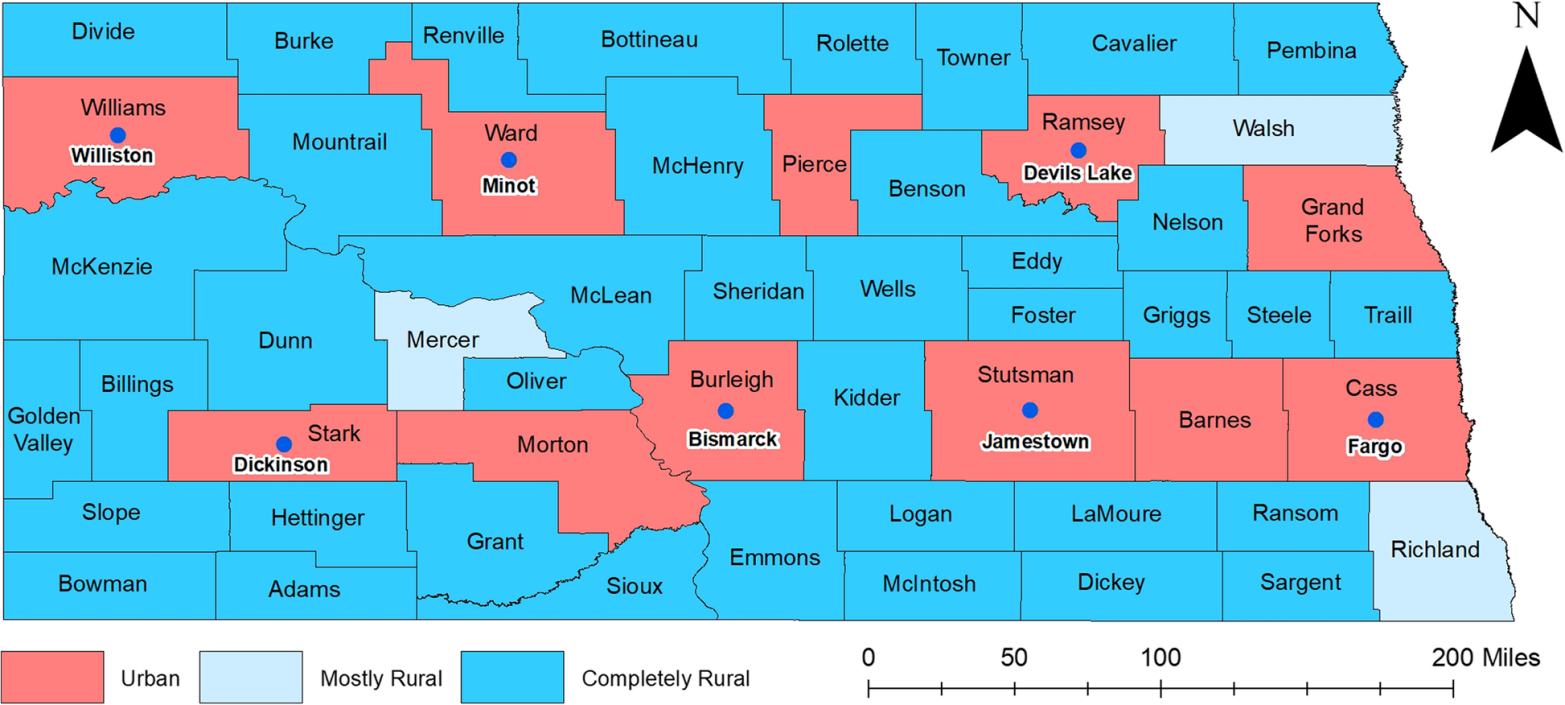
Geographic Distribution of Urban/Rural counties and major cities in North Dakota, USA

The study area included all 53 counties of North Dakota (ND), which has a population of 760,394 comprised of 51% males and 49% females [21]. Geographically, the state lies between 97°-104° W and latitude 45^o^55’-49^o^ N and ranks 19^th^ by area in the United States. As of 2020, the most populated county was Cass county with 179,937 people, while Slope County was the least populated [21]. The racial composition is 86.9% White, 3.4% African American, 5.6% American Indian, and 1.7% Asian. Although 97% of North Dakota’s land is mainly rural, only 39.4% of the total population lives in rural areas [22]. A total of 39 of the 53 counties are classified as completely rural, 3 counties are mostly rural, and 11 counties are urban (Fig. 1).

### Data source and preparation

This retrospective study used secondary data that included confirmed COVID-19 cases reported from March 2020 to September 2021 and obtained from North Dakota Department of Health and Human Services (NDDHHS). Data of total number of COVID-19 tests [Polymerase Chain Reaction (PCR) or Antigen (Ag)] performed in North Dakota during the study period were downloaded from the North Dakota COVID-19 Dashboard [23]. County-level percentages of positive tests were computed and expressed as number of positive COVID-19 PCR tests per 100 tests. County-level incidence risks were computed based on confirmed COVID-19 cases reported during the study period and expressed as number of cases per 100,000 population. The 5-year population estimates for the time period 2015–2019, used as the denominator for calculating county level COVID-19 incidence risks, were obtained from the American Community Survey [21]. County-level cartographic boundary file was downloaded from the United States Census Bureau TIGER Geodatabase [24] and used for all spatial displays.

### Temporal and geographical distribution of COVID-19

Descriptive statistics of percentages of positive tests and COVID-19 incidence risks were calculated using SAS 9.4 [25]. To assess the changes in geographic disparities over time, percentages of positive tests and incidence risks were computed for four time periods (March 2020-July 2020, August 2020-December 2020, January 2021-July 2021, and August 2021-September 2021). Peak periods (August-December 2020 and August–September 2021) were classified as those with incidence risks ≥ 500 per 100,000 population in a month otherwise they were considered non-peak periods (March-July 2020 and January-July 2021). Temporal changes in incidence risks over the four time periods were displayed graphically in Microsoft Excel [26].

County-level Spatial Empirical Bayesian (SEB) smoothed incidence risks were computed in GeoDa [27–29] to adjust for spatial autocorrelation and small number of cases/population sizes of some counties.

### Spatial clusters detection method

Tango’s flexible spatial scan statistic (FSSS) was computed in FleXScan [30] to identify counties with significantly high percentages of positive tests and COVID-19 incidence risks [31]. Scanning for spatial clusters was done using a maximum spatial scanning window of 15 counties specifying restricted log likelihood ratio (LLR) and an alpha of 0.2. A critical p-value of 0.05 and 999 Monte Carlo simulations were used to identify the statistically significant clusters. Potential clusters were ordered based on their restricted LLR. The cluster with the largest value of the restricted LLR was considered the primary cluster. Only high-risk clusters with relative risks ≥ 1.10 were considered meaningful.

### Cartographic display

All cartographic displays were performed in ArcGIS version 10.8.1 [32]. Choropleth maps, generated in ArcGIS version 10.8.1 (ESRI) [32], were used to visualize the distribution of percentages of positive tests and both unsmoothed and smoothed COVID-19 incidence risks using Jenk’s optimization classification scheme. The choropleths maps were generated for the four-time periods; March 2020-July 2020, August 2020-December 2020, January 2021-July 2021, and August 2021-September 2021. Identified high risk spatial clusters were also displayed using ArcGIS.

## Results

### Spatial distribution

**Fig. 2:**
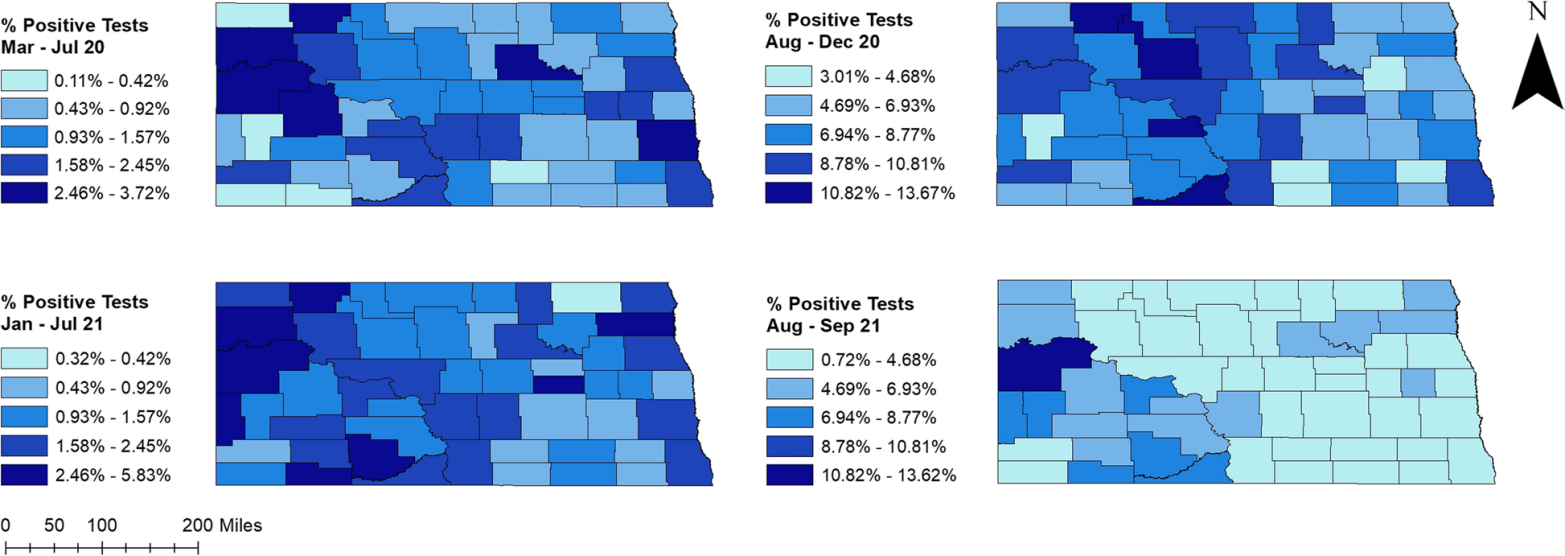
Distribution of positive COVID-19 PCR tests per 100 tests in North Dakota from March 2020 to September 2021

**Fig. 3:**
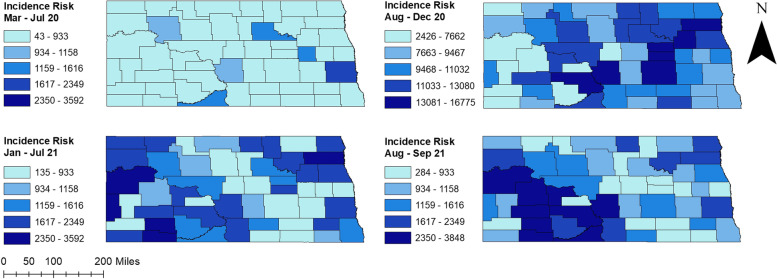
Distribution of unsmoothed COVID-19 incidence risks per 100,000 population in North Dakota from March 2020 to September 2021

**Fig. 4:**
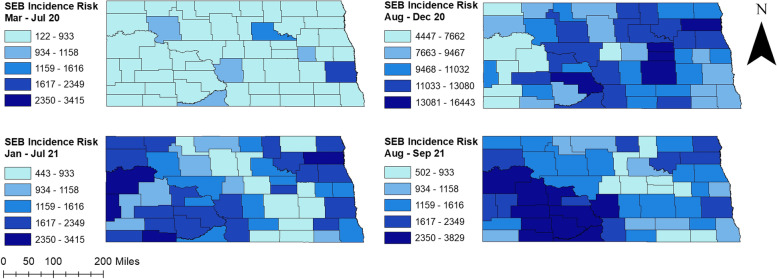
Distribution of SEB smoothed COVID-19 incidence risks per 100,000 population in North Dakota from March 2020 to September 2021

Percentages of positive tests varied across the state ranging from 0.11% to 13.67%. Higher percentages of positive tests were observed in August-December 2020 and August–September 2021 (0.72%-13.67%) compared to March-July 2020 and January-July 2021 (0.11%-5.83%) (Fig. 2). Counties located in the western part of the state had consistently high percentages of positive tests during peak and non-peak periods. Several counties in the easternmost part of the state, on the other hand, had high percentages of positive tests in non-peak periods. Additionally, high percentages of positive tests were observed in central ND counties from March 2020 to July 2021. More than half of the counties had ≥ 5% positive tests in August-December 2020 (Fig. 2).The total number of COVID-19 confirmed cases over the study period was 117,617. The spatial patterns in the unsmoothed maps (Fig. 3) were not as apparent as those in the smoothed maps (Fig. 4). The county-level SEB incidence risks varied by geographical region and ranged from 122 to 16,443 cases per 100,000 population (Fig. 4). Higher risks were observed in August-December 2020 and August–September 2021 whereas the lowest risks in most counties were observed during March-July 2020. The higher incidence risk tends to occur in the middle and southwestern parts of the state. It is also worth noting that most of the counties with high incidence risks were located in rural areas although some urban counties (Cass, Burleigh, and Morton) also had high incidence risks.

### Clusters of COVID-19 incidence risks

**Fig. 5:**
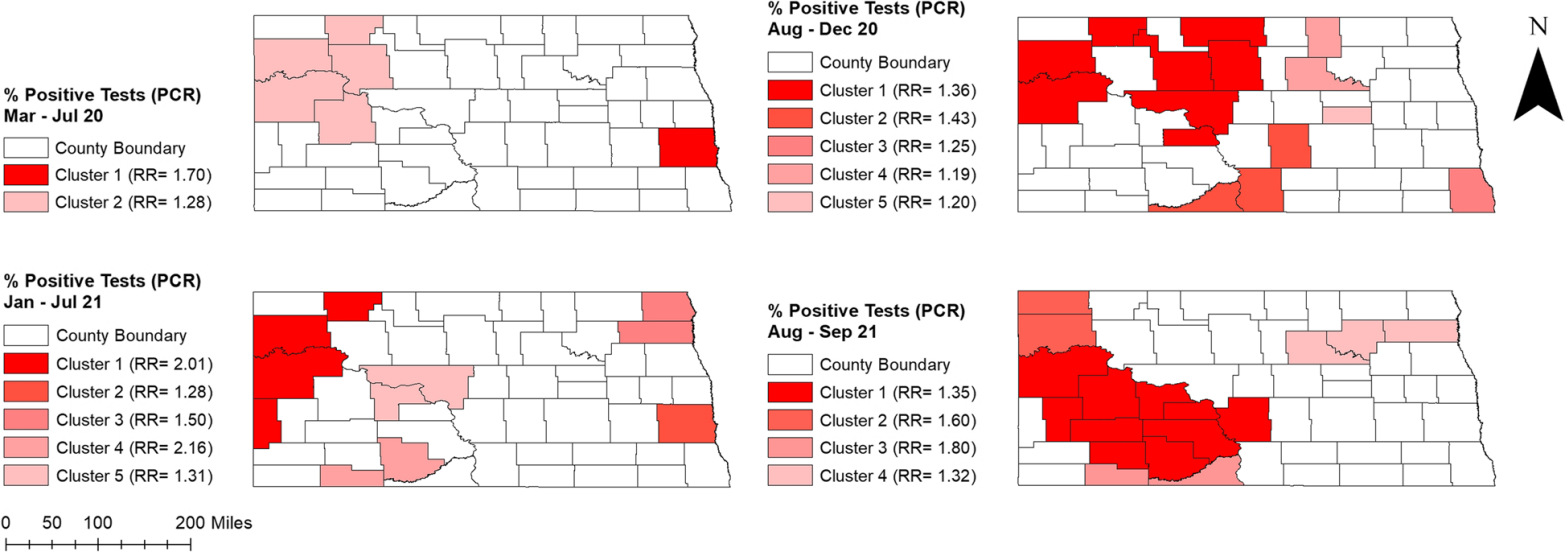
High-risk spatial clusters of percentage of positive COVID-19 tests identified in North Dakota from March 2020 to September 2021

Similar to the geographic distribution of percentages of positive COVID-19 tests, significant clusters of high percentages of positives tests were consistently identified in the western part of the state (Figs. 2 and 5). However, a few small clusters of high percentages of positive tests detected in August-December 2020 were located in the central part of state (Table 1, Fig. 5).

**Table 1:** Purely spatial clusters of high percentages of positive COVID-19 tests identified in North Dakota from March 2020-September 2021

**Period**	**Cluster**	**Total no. of tests**	**Observed positive tests**	**Expected positive tests**	**No. of counties**	**RR**^a^	***p*-value**
March 2020-July 2020	Cluster 1	81,928	2,925	1,716	1	1.70	0.001
	Cluster 2	17,354	465	364	5	1.28	0.001
August 2020-December 2020	Cluster 1	136,239	14,648	10,791	8	1.36	0.001
	Cluster 2	9,949	1,125	788	3	1.43	0.001
	Cluster 3	14,276	1,408	1,131	1	1.25	0.001
	Cluster 4	11,920	1,121	944	2	1.19	0.001
	Cluster 5	5,232	499	414	1	1.20	0.020
January 2021-July 2021	Cluster 1	39,304	1,351	673	4	2.01	0.001
	Cluster 2	155,799	3,419	2,667	1	1.28	0.001
	Cluster 3	15,805	406	271	2	1.50	0.001
	Cluster 4	3,280	121	56	2	2.16	0.001
	Cluster 5	12,548	281	215	2	1.31	0.005
August 2021-September 2021	Cluster 1	106,696	6,148	4,546	10	1.35	0.001
	Cluster 2	9,416	641	401	2	1.60	0.001
	Cluster 3	2,147	165	92	2	1.80	0.001
	Cluster 4	8,904	499	379	3	1.32	0.001

^a^Relative Risk

**Fig. 6:**
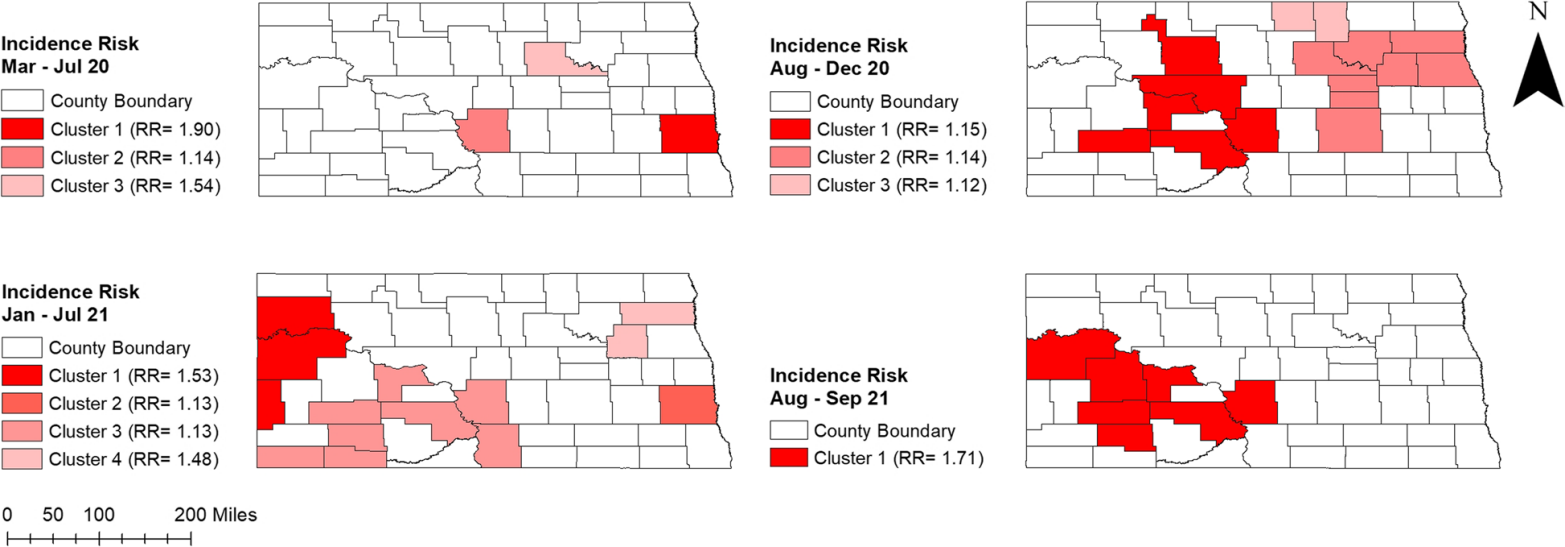
High-risk spatial clusters of COVID-19 incidence risks identified in North Dakota from March 2020 to September 2021

Significant high-risk spatial clusters of COVID-19 incidence were identified in the middle and southern-west parts of the state (Fig. 6), which were consistent with the spatial distribution of COVID-19 incidence risks (Figs. 3 and 4). The number of counties involved in the spatial clusters increased between 2020 and 2021 (Table 2, Fig. 6). During August-December 2020, two high-risk clusters were detected. The primary cluster with a relative risk (RR) of 1.15 was identified in central North Dakota, containing six counties (Fig. 6). A high-risk spatial cluster with relative risk 1.71 was also detected in the central and western part during August 2021-September 2021. This cluster included seven counties and, except for inclusion of Ward and Mclean counties, was generally similar to the primary cluster found in August 2020-December 2020 time period. On the other hand, a primary cluster with only three counties (McKenzie, Williams, Golden Valley) was detected in the middle and western part during January 2021-July 2021. Furthermore, two secondary clusters were identified in the Eastern part of the state during August-December 2020. Interestingly, no secondary cluster was detected in the August–September 2021 time period.

**Table 2:** Purely spatial clusters of high COVID-19 incidence risk identified in North Dakota from March 2020-September 2021

**Period**	**Cluster**	**Population**	**Observed cases**	**Expected cases**	**No. of counties**	**RR**^a^	***p*-value**
Mar 2020-Jul 2020	Cluster 1	176,975	2,925	1,540	1	1.90	0.001
	Cluster 2	94,793	944	825	1	1.14	0.008
	Cluster 3	6,873	92	60	1	1.54	0.035
Aug 2020-Dec 2020	Cluster 1	243,600	30,519	26,613	6	1.15	0.001
	Cluster 2	128,851	16,033	14,077	8	1.14	0.001
	Cluster 3	16,735	2,049	1,828	2	1.12	0.001
Jan 2021-Jul 2021	Cluster 1	50,387	1,309	858	3	1.53	0.001
	Cluster 2	176,975	3,419	3,014	1	1.13	0.001
	Cluster 3	176,520	3,407	3,006	8	1.13	0.001
	Cluster 4	13,645	345	232	2	1.48	0.001
Aug 2021-Sep 2021	Cluster 1	185,480	6,073	3,542	7	1.71	0.001

^a^Relative Risk

### Temporal pattern

**Fig. 7:**
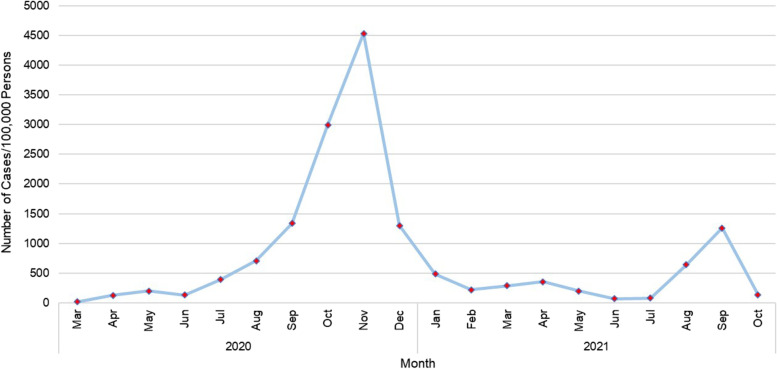
Temporal pattern of COVID-19 incidence risk from March 2020 through September 2021

The overall number of COVID-19 cases increased from 2020 to 2021. Two peaks of incidence risk were observed: (i) August-December 2020 and (ii) August–September 2021 (Fig. 7). The highest risk, 4500 cases per 100,000 persons was identified in the month of November 2020. The two non-peak periods were March-June 2020, and January-July 2021 which showed risks less than 500 cases per 100,000 population.

## Discussion

This study investigated geographic disparities and temporal patterns of county-level COVID-19 incidence risks in North Dakota. The findings of this study are useful for identifying communities with high COVID-19 incidence risks so as to guide planning and intervention efforts.

The observed high risks in August to December 2020 followed by a decline in the Summer, and then a steady increase in August to September 2021 are comparable to findings reported by the Centers for Disease Control and Prevention (CDC) and other previous studies in the US [33–35]. This might be due to the fact that temperature and humidity played a role in human behavioral patterns and viral survival, which favored severe acute respiratory syndrome coronavirus-2 (SARS-CoV-2) transmission [36–39]. Vaccination could be another explanation for the observed temporal pattern since the Food and Drug Administration (FDA) first authorized the COVID-19 vaccine on December 10^th^ 2020, and mass vaccination throughout the US was started a few days later [40].

There was evidence of geographical disparities in COVID-19 incidence risks with most of the high risks being observed in rural communities. This is probably due to high rates of poverty, smoking, respiratory diseases, high blood pressure, and obesity in the rural areas compared to urban areas [41]. Some previous studies also found that behavioral and demographic factors such as smoking history and co-morbidities are associated with COVID-19 incidence [42–46]. The observed high risk in rural areas may also be due to lower healthcare resources since rural areas tend to have lower access to health facilities compared to urban areas [47].

The high incidence risks and high-risk clusters observed in the central and south-western parts of the state may be due to low levels of education attainment in these areas. Statistics show that the percentage of post-secondary degree attainment is substantially higher in the eastern regions compared to the west and central parts of North Dakota [48]. A recent study conducted by Das et al. in St. Louis, Missouri reported a significant association between COVID-19 incidence risk and higher education [49]. This may be closely related to the abilities of individuals with lower levels of education to observe preventive measures such as social distancing. Compared to individuals with lower levels of education, those with high levels of education are more likely to work from home and observe social distancing and hence reduce chances of infection with COVID-19 [50, 51]. Geographical differences in vaccination coverage might be another reason for these disparities. The percentage of the population that received at least one dose of COVID-19 vaccine was comparatively higher in the eastern regions compared to the west and central [52]. Lack of health insurance coverage among the residents of central and south-western parts of North Dakota might be a reason for the lower number of vaccinations. There is evidence that health insurance coverage for individuals under 65 years old was significantly higher in the eastern counties compared to the other counties of North Dakota [53]. Although COVID-19 immunizations are free to all, it might be possible that uninsured individuals may not understand that COVID-19 vaccination is free for all.

Racial differences in population distribution may also account for the observed disparities. A previous study reported that minority populations tend to work in high-contact occupations (hotel, restaurant, road construction, and food service), which increases the probability of SARS-CoV-2 infection [54]. Similarly, some previous studies also documented that minority race is associated with low household income, which is significantly associated with COVID-19 risk [55–58]. It is also possible that the unequal distribution of health facilities, distrust by some minority populations, and discrimination may play a role in the observed disparities. Geographical differences in COVID risks among male and female populations might be another reason of these observed disparities. There is evidence that counties with high percentages of females are located in the central and southwest parts of ND [59]. Furthermore, according to NDDHHS, higher risks of COVID-19 was detected among females (52.7%) than males (47.3%) [60]. Similarly, a recently published article reported that counties with more females had higher rates of COVID-19 cases and deaths [61]. This might be due to the fact that two thirds of the workers in the frontline industries (healthcare, childcare and social service, pharmacy technicians, cashier, and customer service representatives) during the pandemic were female [62] and hence they were at higher risk of exposure and infection.

The high percentage of COVID-19 positive tests, as well as high risk clusters of the positive tests, also showed a similar distribution. The western parts of the state tended to have high percentages of positive tests compared to the other parts of North Dakota. This might be due to the high proportions of Hispanic population in these areas [63]. Hispanic people tend to live in high deprivation areas with low access to health care facilities. A previous study conducted by Lewis *et. al.* in Utah reported that the percentage of test positivity increased with the level of deprivation [64].

### Strengths and weaknesses

This is the first study in North Dakota that has investigated geographical disparities of COVID-19 incidence risks using a rigorous spatial statistics approach. The strengh of Tango’s FSSS used in this study is that it does not involve multiple comparisons and can identify both circular and irregularly-shaped clusters. The findings of this study are helpful for guiding resource allocation for control efforts. The current study revealed geographic disparities across North Dakota from March 2020 to September 2021. Performing such investigations on a regular basis will be beneficial in identifying if the geographic distribution of high risk clusters are consistent or if they change over time. Therefore, such investigations should be part of regular health surveillance programs to guide resource allocation geared at reducing disparities. However, this study is not without limitations. It used administrative/surveillance data which may inherently have geographic differences in case attainment and reporting. Although the SEB smoothed rates improved visualization of spatial patterns, they can only be used for visualization and should not be used for statistical analyses and inference. Additionally, although Tango’s FSSS can identify non-circular clusters more accurately, it has low power for detecting circular clusters. Furthermore, the study only investigated disparities at the county level which may not reveal lower level geographic disparities.

## Conclusion

There is evidence of geographic disparities of COVID-19 incidence risks in North Dakota with high risk clusters being observed in the rural central and southwest parts of the state. The findings of this study will be useful in guiding health equity programs aimed at reducing disparities in the burden of COVID-19 in North Dakota. These investigations should be part of regular health surveillance programs to provide the most current information to guide health planning and service provision. Future studies will investigate predictors of the identified disparities to guide planning, disease control and health policy.

## Supplementary Information

**Additional file 1. **The data used in this study have been provided as supporting information files together with the submission.

## Data Availability

All data generated or analyzed during this study are included in this published article and its supplementary information files.

## References

[1] World Health Organization. WHO Director-General’s opening remarks at the media briefing on COVID-19 - 11 March 2020. https://www.who.int/director-general/speeches/detail/who-director-general-s-opening-remarks-at-the-media-briefing-on-covid-19---11-march-2020. Accessed 10 Mar 2022.

[2] World Health Organization. United States of America: WHO Coronavirus Disease (COVID-19) Dashboard With Vaccination Data | WHO Coronavirus (COVID-19) Dashboard With Vaccination Data. https://covid19.who.int/region/amro/country/us. Accessed 10 Mar 2022.

[3] North Dakota health officials confirm 4 more cases of COVID-19; 2 in Burleigh County | Health | bismarcktribune.com. https://bismarcktribune.com/news/local/health/north-dakota-health-officials-confirm-more-cases-of-covid-/article_b1a73e22-7c7f-5cad-ad6c-3ea850e6bea2.html. Accessed 25 Mar 2022.

[4] North Dakota Department of Health and Human Services. Burgum requests major presidential disaster declaration for response to COVID-19 pandemic | Department of Health. https://www.health.nd.gov/news/burgum-requests-major-presidential-disaster-declaration-response-covid-19-pandemic. Accessed 25 Mar 2022.

[5] Fadl N, Ali E, Salem TZ (2021). COVID-19: Risk Factors Associated with Infectivity and Severity. Scand J Immunol.

[6] Garg S, Kim L, Whitaker M, O’Halloran A, Cummings C, Holstein R (2020). Hospitalization Rates and Characteristics of Patients Hospitalized with Laboratory-Confirmed Coronavirus Disease 2019 — COVID-NET, 14 States, March 1–30, 2020. MMWR Morb Mortal Wkly Rep.

[7] Gao Y, Ding M, Dong X, Zhang J, Kursat Azkur A, Azkur D, et al. Risk factors for severe and critically ill COVID‐19 patients: A review. Allergy. 2021;76:428–55. 10.1111/all.14657. 10.1111/all.14657 33185910

[8] Bialek S, Boundy E, Bowen V, Chow N, Cohn A, Dowling N (2020). Severe Outcomes Among Patients with Coronavirus Disease 2019 (COVID-19) — United States, February 12–March 16, 2020. MMWR Morb Mortal Wkly Rep.

[9] Akanbi MO, Rivera AS, Akanbi FO, Shoyinka A. An Ecologic Study of Disparities in COVID-19 Incidence and Case Fatality in Oakland County, MI, USA, During a State-Mandated Shutdown. 10.1007/s40615-020. 10.1007/s40615-020-00909-1 PMC7595050 33124003

[10] Chen JT, Krieger N (2021). Revealing the unequal burden of COVID-19 by income, race/ethnicity, and household crowding: US county versus zip code analyses. J Public Heal Manag Pract.

[11] Mude W, Oguoma VM, Nyanhanda T, Mwanri L, Njue C (2021). Racial disparities in COVID-19 pandemic cases, hospitalisations, and deaths: A systematic review and meta-analysis. J Glob Health.

[12] American Press Media Research Lab. Color of Coronavirus: COVID-19 deaths analyzed by race and ethnicity. https://www.apmresearchlab.org/covid/deaths-by-race. Accessed 25 Mar 2022.

[13] Knittel C, Ozaltun B. What Does and Does Not Correlate with COVID-19 Death Rates. Natl Bur Econ Res. 2020;27391.10.3386/w27391.

[14] Xiong C, Hu S, Yang M, Luo W, Zhang L (2020). Mobile device data reveal the dynamics in a positive relationship between human mobility and COVID-19 infections. Proc Natl Acad Sci U S A.

[15] Zhu Z, Xu S, Wang H, Liu Z, Wu J, Li G, et al. COVID-19 in Wuhan: sociodemographic characteristics and hospital support measures associated with the immediate psychological impact on healthcare workers. Eclinicalmedicine. 2020;24:100443. 10.1016/J.ECLINM.2020.100443. 10.1016/j.eclinm.2020.100443 PMC7311903 32766545

[16] Jia JS, Lu X, Yuan Y, Xu G, Jia J, Christakis NA (2020). Population flow drives spatio-temporal distribution of COVID-19 in China. Nature.

[17] Badr HS, Du H, Marshall M, Dong E, Squire MM, Gardner LM (2020). Association between mobility patterns and COVID-19 transmission in the USA: a mathematical modelling study. Lancet Infect Dis.

[18] Chang S, Pierson E, Koh PW, Gerardin J, Redbird B, Grusky D (2021). Mobility network models of COVID-19 explain inequities and inform reopening. Nature.

[19] Dowd JB, Andriano L, Brazel DM, Rotondi V, Block P, Ding X (2020). Demographic science aids in understanding the spread and fatality rates of COVID-19. Proc Natl Acad Sci U S A.

[20] Esteve A, Permanyer I, Boertien D, Vaupel JW (2020). National age and coresidence patterns shape COVID-19 vulnerability. Proc Natl Acad Sci U S A.

[21] American Community Survey (ACS). https://www.census.gov/programs-surveys/acs. Accessed 3 Aug 2022.

[22] Health and Healthcare in Frontier Areas Overview - Rural Health Information Hub. https://www.ruralhealthinfo.org/topics/frontier. Accessed 3 Aug 2022.

[23] North Dakota Department of Health and Human Services. North Dakota Coronavirus Cases. https://www.health.nd.gov/diseases-conditions/coronavirus/north-dakota-coronavirus-cases. Accessed 21 Sep 2022.

[24] TIGER/Line Geodatabases. https://www.census.gov/geographies/mapping-files/time-series/geo/tiger-geodatabase-file.html. Accessed 9 May 2022.

[25] SAS Institute inc. SAS version 9.4. Cary, NC (2017). https://www.sas.com/en_us/software/stat.html. Accessed 15 May 2022.

[26] Microsoft Corporation. Microsoft Excel. Redmond, WA (2022). https://www.microsoft.com/en-us/microsoft-365/excel. Accessed 3 Aug 2022.

[27] Odoi A, Martin SW, Michel P, Holt J, Middleton D, Wilson J (2003). Geographical and temporal distribution of human giardiasis in Ontorio. Canada Int J Health Geogr.

[28] Haddow AD, Odoi A (2009). The incidence risk, clustering, and clinical presentation of La Crosse virus infections in the Eastern United States, 2003-2007. PLoS One..

[29] Bernardinelli L, Montomoli C (1992). Empirical bayes versus fully bayesian analysis of geographical variation in disease risk. Stat Med.

[30] FleXScan: Software for the Flexible Scan Statistics. https://sites.google.com/site/flexscansoftware/download_e. Accessed 3 Aug 2022.

[31] Tango T, Takahashi K (2005). A flexibly shaped spatial scan statistic for detecting clusters. Int J Health Geogr.

[32] ArcGIS. Introducing ArcGIS Enterprise 10.8.1. https://www.esri.com/arcgis-blog/products/arcgis-enterprise/announcements/arcgis-enterprise-10-8-1/. Accessed 15 May 2022.

[33] Center for Disease Control and Prevention. CDC COVID Data Tracker: Daily and Total Trends. https://covid.cdc.gov/covid-data-tracker/#trends_totalcases_7daycasesper100k_00. Accessed 16 Aug 2022.

[34] Leidman E, Duca LM, Omura JD, Proia K, Stephens JW, Sauber-Schatz EK (2021). COVID-19 Trends Among Persons Aged 0–24 Years — United States, March 1–December 12, 2020. MMWR Morb Mortal Wkly Rep.

[35] Tan AX, Hinman JA, Abdel Magid HS, Nelson LM, Odden MC. Association Between Income Inequality and County-Level COVID-19 Cases and Deaths in the US. JAMA Netw Open. 2021;4:e218799. 10.1001/jamanetworkopen.2021.8799. 10.1001/jamanetworkopen.2021.8799 PMC8094008 33938935

[36] Al Huraimel K, Alhosani M, Kunhabdulla S, Stietiya MH (2020). SARS-CoV-2 in the environment: Modes of transmission, early detection and potential role of pollutions. Sci Total Environ.

[37] Bontempi E, Vergalli S, Squazzoni F (2020). Understanding COVID-19 diffusion requires an interdisciplinary, multi-dimensional approach. Environ Res..

[38] Courtemanche C, Garuccio J, Le A, Pinkston J, Yelowitz A (2020). Strong social distancing measures in the united states reduced the covid-19 growth rate. Health Aff.

[39] Tammes P (2020). Social distancing, population density, and spread of COVID-19 in England: A longitudinal study. BJGP Open.

[40] F.D.A. Panel Gives Green Light to Pfizer’s Covid Vaccine - The New York Times. https://www.nytimes.com/2020/12/10/health/covid-vaccine-pfizer-fda.html. Accessed 16 Aug 2022.

[41] Center for Disease Control and Prevention. About Rural Health | CSELS | OPHSS | CDC. https://www.cdc.gov/ruralhealth/about.html. Accessed 17 Aug 2022.

[42] Igoe M, Das P, Lenhart S, Lloyd AL, Luong L, Tian D (2022). Geographic disparities and predictors of COVID-19 hospitalization risks in the St. Louis Area, Missouri (USA). BMC Public Health..

[43] Coccia M (2021). How do low wind speeds and high levels of air pollution support the spread of COVID-19?. Atmos Pollut Res.

[44] Bansal M (2020). Cardiovascular disease and COVID-19. Diabetes Metab Syndr Clin Res Rev.

[45] Khan MM, Roberson S, Reid K, Jordan M, Odoi A (2021). Geographic disparities and temporal changes of diabetes prevalence and diabetes self-management education program participation in Florida. PLoS ONE.

[46] Khan MM, Odoi A, Odoi EW (2023). Geographic disparities in COVID-19 testing and outcomes in Florida. BMC Public Health.

[47] Cosby AG, Maya McDoom-Echebiri M, James W, Khandekar H, Brown W, Hanna HL (2019). Growth and persistence of place-based mortality in the United States: The rural mortality penalty. Am J Public Health.

[48] The Demographic Statistical Atlas of the United States - Statistical Atlas. https://statisticalatlas.com/state/North-Dakota/Educational-Attainment#data-map/county. Accessed 18 Aug 2022.

[49] Das P, Igoe M, Lenhart S, Lanzas C, LIoyd AL, Odoi A (2022). Geographic Disparities and Determinants of COVID-19 Incidence Risk in the Greater St. Louis Area, Missouri 2021. PLoS One.

[50] Patwary AL, Khattak AJ. Interaction Between Information and Communication Technologies and Travel Behavior: Using Behavioral Data to Explore Correlates of the COVID-19 Pandemic. Transp Res Rec J Transp Res Board. 2022;036119812211166. 10.1177/03611981221116626.

[51] Patwary AL, Khattak AJ. Crash harm before and during the COVID-19 pandemic: Evidence for spatial heterogeneity in Tennessee. Accid Anal Prev. 2023;183:106988. 10.1016/j.aap.2023.106988. 10.1016/j.aap.2023.106988 PMC9874053 36724654

[52] North Dakota Department of Health and Human Services. COVID19 Vaccine Dashboard | Department of Health. https://www.health.nd.gov/covid19vaccine/dashboard. Accessed 19 Aug 2022.

[53] Health care coverage - Health - North Dakota Compass. https://www.ndcompass.org/health/key-measures.php?km=healthcarecoverage#0-6837-g. Accessed 19 Aug 2022.

[54] Wong DWS, Li Y (2020). Spreading of COVID-19: Density matters. PLoS One..

[55] Khubchandani J, Macias Y (2021). COVID-19 vaccination hesitancy in Hispanics and African-Americans: A review and recommendations for practice. Brain, Behav Immun Heal..

[56] Momplaisir FM, Kuter BJ, Ghadimi F, Browne S, Nkwihoreze H, Feemster KA (2021). Racial/Ethnic Differences in COVID-19 Vaccine Hesitancy among Health Care Workers in 2 Large Academic Hospitals. JAMA Netw Open.

[57] Smith AC, Woerner J, Perera R, Haeny AM, Cox JM. An Investigation of associations between race, ethnicity, and past experiences of discrimination with medical mistrust and COVID-19 protective strategies. J Racial Ethn Heal Disparities. 2022;9:1430–42. 10.1007/s40615-021-01080-x . 10.1007/s40615-021-01080-x PMC8195452 34117633

[58] Khan M, Nath ND, Schimidt M, Njau G, Odoi A (2023). Geographic disparities and temporal changes of COVID-hospitalization risks in North Dakota. Front Public Health.

[59] Population - Demographics - North Dakota Compass. https://www.ndcompass.org/demographics/key-measures.php?km=population#0-6815-g. Accessed 12 Jan 2023.

[60] North Dakota Department of Health and Human Services. Coronavirus Cases | Health and Human Services North Dakota. https://www.hhs.nd.gov/north-dakota-coronavirus-cases. Accessed 12 Jan 2023.

[61] McLaughlin JM, Khan F, Pugh S, Angulo FJ, Schmitt HJ, Isturiz RE (2021). County-level Predictors of Coronavirus Disease 2019 (COVID-19) Cases and Deaths in the United States: What Happened, and Where Do We Go from Here?. Clin Infect Dis.

[62] Rho HJ, Brown H, Fremstad S (2020). A basic demographic profile of workers in frontline industries - Center for Economic and Policy Research. Cent Econ policy Res..

[63] The Demographic Statistical Atlas of the United States - Statistical Atlas. https://statisticalatlas.com/state/North-Dakota/Race-and-Ethnicity. Accessed 22 Sep 2022.

[64] Lewis NM, Friedrichs M, Wagstaff S, Sage K, LaCross N, Bui D, et al. Disparities in COVID-19 Incidence, Hospitalizations, and Testing, by Area-Level Deprivation — Utah, March 3–July 9, 2020. MMWR Morb Mortal Wkly Rep. 2020;69:1369–73. 10.15585/mmwr.mm6938a4. 10.15585/mmwr.mm6938a4 PMC7727491 32970656

